# Knowledge, attitude and associated factors towards tuberculosis in Lesotho: a population based study

**DOI:** 10.1186/s12879-019-3688-x

**Published:** 2019-01-29

**Authors:** Tegene Regassa Luba, Shangfeng Tang, Qiaoyan Liu, Simon Afewerki Gebremedhin, Matiko D. Kisasi, Zhanchun Feng

**Affiliations:** 1 School of Medicine and Health Management, Tongji Medical College, Huazhong University of Science and Technology, Hang Kong Road 13, Wuhan, 430030 Hubei China; 2 Ministry of Health, Addis Ababa, Ethiopia; 3 School of Public health, Tongji Medical College, Huazhong University of Science and Technology, Wuhan, China; 4 School of basic medicine, Tongji Medical College, Huazhong University of Science and Technology, Wuhan, China

**Keywords:** Tuberculosis, Knowledge, Attitude, Associated factors, Lesotho

## Abstract

**Background:**

Lesotho has one of the highest rates of tuberculosis (TB) incidence and TB-HIV co-infection in the world. Our study aimed to assess the knowledge, attitude and associated factors towards TB in the general population of Lesotho.

**Methods:**

A cross-sectional analysis from the Lesotho Demographic and Health Survey (LDHS) 2014 was carried out among 9247 respondents. We used the chi-square test as well as univariate and multivariate logistic regression analyses to assess the associations of socio-demographic variables with respondent knowledge of and attitude towards TB.

**Results:**

The overall knowledge of TB in the general population of Lesotho was adequate (59.9%). There was a significant difference between female and male respondents regarding knowledge about TB (67.0% vs. 41.8%). Almost 95% of respondents had “heard of an illness called tuberculosis”, and 80.5% knew that TB can be cured. Only 11.5% knew the correct cause of TB (TB is caused by *Mycobacterium tuberculosis*). Female respondents were relatively aware of TB, knew about the correct cause and mode for transmission of TB and knew that TB is a curable disease compared to male respondents. A higher proportion of respondents (72.8%) had a positive attitude towards TB. Multivariate logistic regression analysis showed that sex (adjusted odds ratio [AOR] = 2.45, 95% CI: 2.10–2.86; *p* < 0.001), age (AOR) =1.76, 95% CI: 1.29–2.41; p < 0.001), educational level (AOR = 6.26, 95% CI: 3.90–10.06; p < 0.001), formerly married or cohabitated (AOR = 1.42, 95% CI: 1.10–1.85; *p* = 0.008), mass media exposure (AOR = 1.33, 95% CI: 1.08–1.64; p = 0.008) and occupation (AOR = 1.20, 95% CI: 1.00–1.44; *p* = 0.049) were strongly associated with respondent knowledge of TB. Sex (AOR = 1.19, 95% CI: 1.01–1.41; *p* = 0.034), educational level (AOR = 1.661, 95% CI: 06–2.60; *p* = 0.028), mass media exposure (AOR = 1.31, 95% CI: 1.06–1.62; *p* = 0.012) and occupation (AOR = 1.26, 95% CI: 1.04–1.52; *p* = 0.016) were strongly associated with respondent attitude towards TB.

**Conclusion:**

Strategies to improve the knowledge of Lesotho’s people about TB should focus on males, young residents, those who are illiterate, those who are unmarried and farmers. Special attention should be given to males, young residents, rural residents, those who are illiterate and farmers to improve their attitude towards TB in Lesotho.

## Background

TB is an infectious bacterial disease caused by *Mycobacterium tuberculosis* (MTB). Although a remarkable achievement has been made toward containing the disease, and an estimated 37 million lives have been saved through effective diagnosis and treatment of TB since 2000, TB remains a major health problem globally [1]. According to a 2015 global health report, TB is a major cause of morbidity and mortality, ranking alongside the human immunodeficiency virus (HIV) as a leading cause of death worldwide [2]. In 2015, 10.4 million people were estimated to have fallen ill with TB, and 1.8 million people died of TB globally [3].

The burden of tuberculosis falls most heavily on developing countries. Among the estimated 1.8 million deaths due to TB, over 95% occurred in low- and middle-income countries in 2015 [2]. Africa is the continent most affected by tuberculosis. From the estimated 1.2 million new HIV-positive TB cases that occurred globally in 2014, almost three-quarters were in the African region [2]. Similarly, in 2015, among the estimated 1.2 million new cases of TB among people who were HIV-positive, 71% were living in Africa [3].

Lesotho is among the 30 highest TB- and TB/HIV-burdened countries, and TB is among the leading causes of morbidity and mortality in Lesotho [3]. Lesotho has one of the highest rates of TB incidence and TB-HIV co-infection in the world. The incidence (including HIV + TB) of TB in 2015 was 788 patients per 100,000 of the population, and 74% of TB patients were co-infected with HIV in 2014 [4, 5]. Lesotho is also one of the countries most highly burdened with multidrug resistant TB (MDR -TB). According to World Health Organization (WHO) estimates of TB and MDR-TB burden, 4.8% of new TB cases and 14% of previously treated TB cases were estimated to have MDR in Lesotho [4].

Few studies have been conducted in Lesotho to examine the knowledge about tuberculosis among different groups of the community [6–10]. At the national level, there have not been any published studies on the knowledge, attitude and associated factors towards TB among the general population of Lesotho. Lack of knowledge of the people and a negative attitude towards TB is one of the major problems in preventing, controlling and ending TB. Thus, we assessed the knowledge, attitude and associated factors towards tuberculosis among the general population of Lesotho.

## Methods

The current study was a cross-sectional analysis from the Lesotho Demographic and Health Survey (LDHS-2014), which was obtained from the United States Agency for International Development demographic health survey (USAID-DHS) program datasets. LDHS-2014 was conducted by the Lesotho Ministry of Health [11].

LDHS-2014 data collection took place from 22 September to 7 December 2014. Data collection was carried out by well-trained data collectors under the close supervision of senior staff members from the ministry of Health of Lesotho. The 2014 LDHS followed a two-stage sample design. In the first stage, 400 clusters (sample points) were selected, 118 in urban areas and 282 in rural areas. The second stage involved systematic sampling of households. A total of 9942 individuals (25 households from each sample point) were randomly selected from the list recorded in July 2014. From the selected households, 9402 individuals were successfully interviewed, yielding a response rate of 99%. In the interviewed households, 6818 eligible women and 3133 eligible men were identified, 6621 women (response rate 97%) and 2931 men (response rate 94%) were successfully interviewed. In general, out of the total 2.2 million people of Lesotho [12], 9552 nationally representative residents participated in the LDHS-2014.

LDHS-2014 was carried out according to the protocol of the Demographic and Health Survey (DHS) program, under the close supervision of the Ministry of Health of Lesotho. The LDHS survey was pretested, and respondents were interviewed by a survey taker to collect data on socio-demographic characteristics and major health indicators, including knowledge, attitudes, and behavior-related issues about HIV/AIDS, tuberculosis, other sexually transmitted infections and non-communicable diseases.

After the relevant variables for the current study were identified, questionnaire answers related to TB awareness and attitudes on the cause, mode of transmission, signs and symptoms, treatment of TB and some key socio-demographic factors were taken from the primary survey dataset. The different datasets for women and men were explored, and in order to have a comparable age distribution, males aged 50–59 years were excluded from the analysis of the current study. Hence, a total of 9247 respondents (6621 women and 2626 men) were included in our analysis. Those respondents missing data in the primary data were excluded.

### Outcome variable

We used the question “Q1: Have you ever heard of an illness called TB?” for the Respondent’s comprehensive TB knowledge. In addition, the following 15 questions were used to evaluate the level of respondent’s knowledge on the cause (4 questions), signs and symptoms (6 questions), mode of transmission (4 questions) and TB treatment (1 question).

Q2: TB is caused by bacteria called *Mycobacterium tuberculosis*?

Q3: TB is caused by cold temperature?

Q4. TB is caused by dust/pollution?

Q5 TB is caused by smoking?

Q6: TB is spread from person to person through the air when coughing or sneezing?

Q7: TB can be transmitted by sharing utensils?

Q8: TB can be transmitted through food?

Q9. TB can be transmitted through sexual contact?

Q10: A person who is infected with TB coughs for several weeks?

Q11: A person who is infected with TB has persistent fever?

Q12: A person who is infected with TB sweats during the night?

Q13: A person who is infected with TB has pain in the chest or back?

Q14: Loss of appetite is one of the symptoms of TB?

Q15: Weight loss is one of the symptoms of TB?

Q16: Can tuberculosis be cured?

The outcome of interest for this analysis was “respondent’s adequate knowledge and positive attitude towards TB”. The respondent’s adequate knowledge on the cause, mode of transmission, signs and symptoms and treatment of TB was defined as “yes” if the respondent correctly answered > 50% (≥ 9 questions) out of the total 16 questions; correct answers were coded as “1”. Incorrect answers were coded as “0” and considered as “misconceptions” for the cause and mode of transmission of TB.

We used two questions to assess the respondent’s attitude towards TB (Q1: Would you be willing to work with someone previously treated for TB? and Q2: Would you want a family member’s TB to be kept secret?). The overall respondents attitude towards TB was defined as “positive acceptance” if the respondent correctly answered both questions (“yes” for the first question and “no” for the second question).

### Independent variables

In the current study, the independent variables we used to determine the association between respondent’s knowledge and attitude towards TB are sex, age, religion, place of residence, level of education, marital status, wealth index, occupation and exposure to mass media. We categorized them as follows: age (15–24, 25–34, 35–44 & 45–49 years), place of residence (rural vs. urban), educational status (no education, primary, secondary, higher), religion (no religion, Christian, Muslim and other), wealth index (poor, middle, rich), current marital status (never married, currently married/cohabiting and formerly married/cohabiting), media exposures (no, yes) and occupation (agricultural, non-agriculture). Exposure to mass media was defined as watching television, listening radio and reading a newspaper at least once a week for the current study. The wealth index was determined using the primary data and was based on a standard set of household assets, dwelling characteristics, and ownership of consumer items. We re-grouped wealth index and occupation based on the number of respondents to each variable in accordance with the suitability of the analysis for the current study.

### Statistical analysis

The respondent’s background in all categories was checked. A chi-square test was employed to check the statistical significance of the association between socio-economic factors and knowledge of the respondents and their attitude towards TB. All the variables found to be significant (*p* < 0.001) were subjected to logistic regression analysis to recheck the association between socioeconomic factors and knowledge of the respondents as well as their attitude towards TB in a univariate analysis. We also calculated the adjusted odds ratio (AOR, 95% confidence interval CI) and assessed the degree and the consistency of the associations between socioeconomic factors and knowledge and attitude of the respondents towards TB in multivariate regression analysis, controlling for potential confounding between the variables. We checked multi co-linearity between the included variables, and the results (VIF) were < 3 for all. Associations were considered significant at 푝< 0.05. All analyses were conducted using SPSS software version 22.

## Results

Table 1 presents the socio-demographic characteristics of the respondents. Greater proportions of the participants were younger (44%), were from a rural area (67.3%), belonged to a relatively rich household (44.4%), had secondary-level education (46.8%) and were exposed to mass media (78.5%). Nearly half of the respondents were married or cohabited (49.8%), and approximately 96% were Christian. Most respondents were female (71.6%), and more than half of the female respondents (58.5%) had a secondary level of education or higher.

**Table 1:** Distribution of socio-demographic characteristics of 9247 respondents by knowledge of TB and attitude towards TB among the general population of Lesotho

Variables	Female	Male	Total
	6621 (71.6%)	2626 (28.4%)	9247
Age			
15–24	2842 (42.9)	1224 (46.6)	4066 (44.0)
25–34	1979 (29.9)	739 (28.1)	2718 (29.4)
35–44	1310 (19.8)	497 (18.9)	1807 (19.5)
45–49	490 (7.4)	166 (6.3)	656 (7.1)
Type of Place of residence			
Urban	2202 (33.3)	821 (31.3)	3023 (32.7)
Rural	4419 (66.7)	1805 (68.7)	6224 (67.3)
Educational status			
No education	8 (1.2)	37 (9.0)	318 (3.4)
Primary	2665 (40.3)	1228 (46.8)	3893 (42.1)
secondary	3354 (50.7)	972 (37.0)	4326 (46.8)
Higher	521 (7.9)	189 (7.2)	710 (7.7)
Religion			
No religion	65 (1.0)	195 (7.4)	260 (2.8)
Christian	6473 (97.8)	2395 (91.2)	8868 (95.9)
Islam	11 (0.2)	9 (0.3)	20 (0.2)
Other	72 (1.1)	27 (1.0)	99 (1.1)
Wealth index			
Poor	2321 (35.1)	969 (36.9)	3290 (35.6)
Middle	1307 (19.7)	542 (20.6)	1849 (20.0)
Rich	2993 (45.2)	1115 (42.5)	4108 (44.4)
Current Marital Status			
Never Married	2201 (33.2)	1464 (55.8)	3665 (39.6)
Currently Married/Cohabiting	3609 (54.5)	993 (37.8)	4602 (49.8)
Formerly Married/Cohabiting	811 (12.2)	169 (6.4)	980 (10.6)
Media Exposure			
No	1396 (21.1)	590 (22.5)	1986 (21.5)
Yes	5225 (78.9)	2036 (77.5)	7261 (78.5)
Occupation			
Agricultural	305 (11.9)	649 (39.4)	954 (22.7)
Non Agricultural	2255 (88.1)	997 (60.6)	3252 (77.3)

Table 2 presents the correct knowledge of respondents about the disease called TB, its cause, mode of transmission, signs and symptoms, and treatment and related attitudes. The overall knowledge of TB in the general population of Lesotho was adequate (59.9%). However, there was a significant difference between female and male respondents concerning knowledge about TB (67.0% vs. 41.8%, respectively). Out of the total 9247 respondents, 8756 (94.7%) had ever heard (been aware) of the disease called TB, and 7445 (80.5%) knew that TB is a curable disease. A relatively greater proportion of female respondents had awareness of TB and knew that TB is a curable disease compared to male respondents (96.3, 83.3% vs. 90.6, 73.5%).

**Table 2:** Correct Knowledge of respondents on TB awareness, cause, transmission, sign &symptom, treatment and attitudes towards TB in the general population of Lesotho

Variables	Correct knowledge- N (%)		
	Female	Male	Total
	6621 (71.6%)	2626 (28.4%)	9247
TB Awareness			
Heard of tuberculosis or TB (yes)	6377 (96.3)	2379 (90.6)	8756 (94.7)
TB Cause			
Microbes/germs/bacteria (yes)	795 (12.0)	270 (10.3)	1065 (11.5)
Smoking (no)	1597 (24.1)	833 (31.7)	2430 (26.3)
Exposure to Cold temp. (no)	890 (13.4)	333 (12.7)	1223 (13.2)
Dust/Pollution (no)	2767 (41.8)	1327 (50.5)	4094 (44.3)
TB Signs or Symptoms			
Coughing for several weeks (yes)	3877 (58.6)	1259(47.9)	5136 (55.5)
Fever (yes)	537 (8.1)	124 (4.7)	661 (7.1)
Loss of appetite (yes)	1744 (26.3)	360 (13.7)	2104 (22.8)
Night Sweating (yes)	2905 (43.9)	622 (23.7)	3527 (38.1)
Pain in chest or back (yes)	468 (7.1)	197 (7.5)	665 (7.2)
Weight loss (yes)	2984 (45.1)	894 (34.0)	3878 (41.9)
TB Transmission			
Coughing or Sneezing (yes)	5370 (81.1)	1869 (71.2)	7239 (78.3)
Sharing utensils (no)	454 (6.9)	185 (7.0)	639 (6.9)
food	101 (1.5)	64 (2.4)	165 (1.8)
Sexual contact (no)	71 (1.1)	39 (1.5)	110 (1.2)
TB treatment			
TB can be cured (yes)	5514 (83.3)	1931 (73.5)	7445 (80.5)
Adequate knowledge (Correct answers to ≥9 questions)	4438 (67.0%)	1098 (41.8%)	5536 (59.9%)

A great proportion of respondents (78.3%) knew the correct mode of transmission of TB (TB can be spread from person to person through air when coughing or sneezing). However, misconceptions remained high. Only 6.9, 1.8 and 1.2% of respondents knew that TB cannot be spread from person to person by sharing utensils and food or by sexual contact, respectively.

The respondent knowledge of the cause of TB was very low. Out of 9247 respondents, only 1065 (11.5%) knew that the cause of TB is the bacteria called *Mycobacterium tuberculosis*. In addition, only 26.3, 13.2% & 44.3% respondents knew that TB is not caused by smoking, cold temperature and dust/pollution, respectively. Similarly, the respondent’s knowledge about signs and symptoms of TB was low. Coughing for several weeks was selected by most of the respondents (55.5%), followed by weight loss (41.9%), sweating during the night (38.1%), loss of appetite (22.8%), pain in the chest or back (7.2%) and persistent fever (7.1%).

The overall respondent attitude towards TB is presented in Table 3. A higher proportion of respondents (72.8%) had a positive attitude towards TB. Most respondents (93.2%) were willing to work with someone previously treated for TB, and only 21.3% of the respondents said that they “Would keep it a secret from neighbors if a member of their family got tuberculosis”.

**Table 3:** Attitude of respondent’s towards TB in the general population of Lesotho

Variables	Respondent’s attitude towards TB - N (%)		
	Female	Male	Total
Keep secret when family member gets TB (No)	4980 (78.1%)	1891 (75.8%)	6871 (77.4%)
Would be willing to work with someone previously treated for TB (Yes)	5994 (94.0%)	2278 (91.3%)	8272 (93.2%)
Positive acceptance (correct answers to both questions)	4723 (74.1%)	1739 (69.7%)	6462 (72.8%)

Table 4 shows the statistical significance of the association between socio-demographic variables and knowledge of the respondents and their attitude towards TB. All the socio-demographic variables (age, place of residence, educational status, religion, marital status, wealth index, media exposure and occupation) were significantly associated with the respondents’ knowledge about TB and their attitude towards TB (*p* < 0.001).

**Table 4:** Associations between socio–demographic variables and knowledge of respondents about tuberculosis in chi–square test

Variables	Adequate knowledge N (%)	χ^2^	*P*-Value	Positive Attitude N (%)	χ^2^	P-Value
Total	5536 (59.9)			6462 (72.8)		
Age		73.964	< 0.001		90.220	< 0.001
15–24	224 (40.6)			2606 (40.3)		
25–34	1678 (30.3)			1969 (30.5)		
35–44	1165 (21.0)			1395 (21.6)		
45–49	447 (8.1)			492 (7.6)		
Type of Place of residence		114.531	< 0.001		65.671	< 0.001
Urban	2081 (37.6)			2321 (35.9)		
Rural	3455 (62.4)			4141 (64.1)		
Educational status		484.179	< 0.001		104.108	< 0.001
No education	94 (1.7)			190 (2.9)		
Primary	1907 (34.4)			2482 (38.4)		
secondary	2944 (53.2)			3212 (49.7)		
Higher	591 (10.7)			578 (8.9)		
Religion		38.796	< 0.001		6.4996	< 0.090
No religion	102 (1.8)			160 (2.5)		
Christian	5366 (96.9)			6217 (96.2)		
Islam	12 (0.2)			15 (0.2)		
Other	56 (1.0)			70 (1.1)		
Current Marital Status		68.44	< 0.001			
Never Married	2003 (36.2)			2436 (37.7)	22.209	< 0.001
Currently Married/Cohabiting	2878 (52.0)			3293 (51.0)		
Formerly Married/Cohabiting	655 (11.8)			733 (11.3)		
Wealth index		253.894	< 0.001		84.460	< 0.001
Poor	1573 (28.4)			2046 (31.7)		
Middle	1129 (20.4)			1311 (20.3)		
Rich	2834 (51.2)			3105 (48.1)		
Media Exposure		169.86	< 0.001		78.4	< 0.001
No	887 (16.0)			1161 (18.0)		
Yes	4649 (84.0)			5301 (82.0)		
Occupation		154.244	< 0.001		52.855	< 0.001
Agricultural	397 (15.6)			593 (19.3)		
Non Agricultural	2142 (84.4)			2486 (80.79)		

The results of univariate and multivariate logistics regression analysis that were used to recheck the significance of the association, predict the independent effect of the given variables and assess the strength of associations between socio-demographic variables and respondent knowledge about TB are shown in Table 5. In univariate analysis, sex, age, place of residence, educational level, religion, marital status, wealth index, mass media exposure and occupation remained statistically significant with the respondent’s knowledge about TB (*p* < 0.001). Multivariate logistics regression analysis showed that sex (AOR = 2.45, 95% CI: 2.10–2.86; p < 0.001), age (AOR) = 1.76, 95% CI: 1.29–2.41; p < 0.001), educational level (AOR = 6.26, 95% CI: 3.90–10.06; p < 0.001), marital status (AOR = 1.42, 95% CI: 1.10–1.85; *p* = 0.008), mass media exposure (AOR = 1.33, 95% CI: 1.08–1.64; p = 0.008) and occupation (AOR = 1.20, 95% CI: 1.00–1.44; *p* = 0.049) were strongly associated with respondent knowledge about TB. Place of residence and religion were not associated with respondent knowledge about TB, and the association of the wealth index with respondent knowledge about TB did not reach statistically significance in multivariate analysis.

**Table 5:** Associations between socio-demographic variables and knowledge of respondents on TB in univariate and multivariate analyses

Variable	Univariate			Multivariate		
	OR	95% CI	P-Value	AOR	95% CI	P-Value
Sex (Ref = Male)						
Female	2.67	2.42–2.94	< 0.001	2.45	2.10–2.86	< 0.001
Age (Ref = 15–24)						
25–34	1.27	1.14–1.41.	< 0.001	1.08	0.89–1.31	0.423
35–44	1.51	1.34–1.71.	< 0.001	1.40	1.13–1.75	0.003
45–49	1.80	1.50–2.17	< 0.001	1.76	1.29–2.41	< 0.001
Place of Residence (Ref = Rural)						
Urban	0.60	0.54–0.66	< 0.001	0.97	0.82–1.16	0.770
Highest Education level (Ref = No Education)						
Primary	2.17	1.68–2.81	< 0.001	1.58	1.07–2.32	0.021
Secondary	4.48	3.46–5.80	< 0.001	3.46	2.31–5.19	< 0.001
Higher	11.10	8.01–15.37	< 0.001	6.26	3.90–10.06	< 0.001
Religion (Ref = No religion)						
Christian	2.25	1.73–2.92	< 0.001	0.84	0.57–1.24	0.390
Islam	2.55	0.92–7.02	0.070	0.45	0.12–1.75	0.250
Other	1.88	1.15–3.06	0.011	0.92	0.44–1.95	0.837
Marital Status (Ref = Never in Union)						
Currently in union/living with a man	1.37	1.25–1.50	< 0.001	1.23	1.02–1.47	0.027
Formerly in union/living with a man	1.70	1.46–1.99	< 0.001	1.42	1.10–1.85	0.008
Wealth Index (Ref = Poorest)						
Middle	1.62	1.43–1.82	< 0.001	1.20	0.97–1.48	0.090
Rich	2.22	2.01–2.45	< 0.001	1.22	0.99–1.50	0.068
Mass Media (Ref = No)						
Yes	2.00	1.80–2.22	< 0.001	1.33	1.08–1.64	0.008
Occupation (Ref = Agricultural)						
Non Agricultural	2.59	2.22–3.02	< 0.001	1.20	1.00–1.44	0.049

*OR* odds ratio, *AOR* adjusted odds ratio, *CI* confidence interval

Table 6 presents the results of the univariate and multivariate analyses that were used to examine the significance and the degree of association between respondent attitude towards TB and independent variables. Sex, age, place of residence, marital status, wealth index and exposure to mass media remained statistically significant (*p* < 0.001). Religion was not associated with respondent attitude towards TB in both the univariate and multivariate analyses. Sex (AOR = 1.19, 95% CI: 1.01–1.41; *p* = 0.034), age (AOR = 2.13, 95% CI: 1.69–2.70: p < 0.001), educational level (AOR = 1.661, 95% CI: 06–2.60; *p* = 0.028), mass media exposure (AOR = 1.31, 95% CI: 1.06–1.62; *p* = 0.012) and occupation (AOR = 1.26, 95% CI: 1.04–1.52; *p* = 0.016) were strongly associated with respondent attitude towards TB.

**Table 6:** Factors associated with respondent’s attitude towards TB in the general population of Lesotho

Variable	Univariate			Multivariate		
	OR	95% CI	P-Value	AOR	95% CI	P-Value
Sex (Ref = Male)						
Female	1.24	1.12–1.38	< 0.001	1.19	1.01–1.41	0.034
Age (Ref = 15–24)						
25–34	1.38	1.24–1.55	< 0.001	1.51	1.24–1.83	< 0.001
35–44	1.79	1.56–2.04	< 0.001	2.13	1.69–2.70	< 0.001
45–49	1.55	1.28–1.89	< 0.001	1.50	1.11–2.04	0.009
Place of Residence (Ref = Rural)						
Urban	0.65	0.59–0.72	< 0.001	0.82	0.68–0.99	0.035
Highest Education level (Ref = No Education)						
Primary	1.13	0.88–1.45	0.345	1.21	0.84–1.74	0.296
Secondary	1.68	1.31–2.16	< 0.001	1.53	1.04–2.25	0.032
Higher	2.46	1.80–3.35	< 0.001	1.66	1.06–2.60	0.028
Religion (Ref = No religion)						
Christian	1.40	1.07–1.84	0.0145	1.08	0.73–1.58	0.704
Islam	1.95	0.63–6.05	0.250	1.33	0.27–6.45	0.723
Other	1.40	0.83–2.35	0.210	1.71	0.75–3.89	0.205
Marital Status (Ref = Never in Union)						
Currently in union/living with a man	1.21	1.10–1.34	< 0.001	0.95	0.78–1.15	0.582
Formerly in union/living with a man	1.38	1.17–1.63	< 0.001	0.95	0.72–1.24	0.687
Wealth Index (Ref = Poorest)						
Middle	1.36	1.20–1.55	< 0.001	1.02	0.82–1.27	0.868
Rich	1.63	1.47–1.81	< 0.001	0.97	0.78–1.21	0.816
Mass Media (Ref = No)						
Yes	1.64	1.47–1.84	< 0.001	1.31	1.06–1.62	0.012
Occupation (Ref = Agricultural)						
Non Agricultural	1.81	1.54–2.13	< 0.001	1.26	1.04–1.52	0.016

*OR* Odds ratio, *AOR* adjusted odds ratio, *CI* confidence interval

## Discussion

Most of the study participants (94.7%) had heard about the disease called tuberculosis, which is similar to studies conducted in Nigeria, India and Pakistan [13–17]. In addition, the overall knowledge of respondents about TB was adequate (59.9%). However, the overall knowledge of male respondents about TB was very low (41.8%). The female respondents had adequate knowledge (67.0%) about TB and were relatively more knowledgeable about the cause, signs and symptoms, mode of transmission and treatment of TB compared to male respondents. This could be due to the respondent’s level of education. Among all female respondents, 58.5% had a secondary level of education or above, while only 44.2% of male respondents did; out of all female respondents, only 1.2% had no education, whereas 9.0% of male respondents had no education.

Most respondents knew that TB is a curable disease and what was the correct mode of transmission of TB. Approximately 80.5% of respondents knew that TB is a curable disease, and 78.3% of them answered correctly to the question “TB can spread from person to person through the air when coughing or sneezing?” This finding is consistent with studies from Brazil, India and Tanzania [18–20]. The respondent’s knowledge of the cause of TB was very low. The findings of the current study agreed with studies conducted in Afar, Somali and the Gambella regional states of Ethiopia [21–23]. However, a study conducted on the knowledge and awareness of tuberculosis among students in Malawi, Ethiopia and India indicated that 90, 81.7 and 81% of the respondents, respectively, knew that TB is caused by a bacterial infection [24–26]. This may indicate that education is important to improve the knowledge of people about TB, in general, and its cause, in particular. The knowledge of respondents about the signs and symptom of TB was also low. Coughing for several weeks was answered correctly by 55.5% of respondents followed by weight loss (41.9%), sweating during night time (38.1%), loss of appetite (22.8%), pain in chest or back (7.2%) and persistent fever (7.1%). However, a study conducted in Maseru, Lesotho, indicating that more than 90% of its respondents correctly identified the symptoms of TB was not consistent with this study [9].

Educational level and age were strongly associated with the respondent knowledge about TB. The study participants who had higher educational levels were 6.26 more likely to be aware of TB than those who had no education, and respondents in the category of 45–49 years old were 1.76 times more likely to have knowledge of TB than the youngest age group (15–24 years). Our findings are consistent with studies from Nigeria, Ethiopia and Uganda [27–29]. Currently in union and formerly in union (*p* = 0.027/0.008), media exposure (*p* = 0.008) and occupation (*p* < 0.049) were also positively associated with the respondent’s knowledge of TB. The respondents currently married/cohabitating, formerly married/cohabitating and from non-agricultural households were 1.23, 1.42 and 1.20 times more likely to have better knowledge of TB, respectively, compared to those who were never married/cohabitating and in agricultural households, respectively. The potential explanation is that people who were married had access to mass media and more chances to discuss TB with each other. Similarly, those who belonged to non-agriculture households had more access to mass media and education compared to respondents who were farmers. The respondents from nonagricultural household were also 1.26 times more likely to have a positive attitude towards TB.

The respondents who had access to mass media were 1.33 times more likely to have adequate knowledge about TB and 1.31 times more likely to have positive attitude towards TB compared to those who did not have media exposure. From these results, we may explain that media can play a pivotal role in improving the general knowledge of people about TB and their attitude towards TB. The previous study conducted in Maseru, Lesotho, also reported that media was the main source of TB information [9].

Both female and male respondents had a positive attitude towards TB. The studies conducted in Croatia, Lusaka (Zambia), Bangladesh and Nigeria [27, 30–32] were consistent with the current study. Females, adults in the age group of 33–44 and respondents who had media exposure were 1.19, 2.13 and 1.3 times more likely, respectively, to have a positive attitude towards TB compared to males, younger respondents in the age group of 15–24 and those who had no media exposure.

We believe that our study has some limitations. First, we did not evaluate the respondents’ knowledge about methods of preventing tuberculosis and their knowledge about TB-HIV co-infection. Second, some variables that may have an effect on people’s knowledge and attitudes towards TB, such as the distance to reach a health facility, the time required to get health services and regional category, are not analyzed in our study. The TB knowledge questionnaire in the primary dataset likewise did not incorporate some important questions. For instance, the question “drinking unsterilized milk can cause TB?” is not included in the primary data. Hence, we could not analyze it in the current study. Third, due to the lack of similar studies conducted in Lesotho, we are limited in our ability to refer to past studies from the country in our discussion of the present study. Lastly, as it was a cross-sectional study, it is not possible to determine the cause and effect relationships of the associations.

To the best of our knowledge, this study was the first to evaluate the respondent’s knowledge, attitude and associated factors towards TB using a nationally representative sample in Lesotho. Hence, the findings of this study would contribute greatly to interventions aimed at increasing awareness and developing a positive attitude in the general population regarding TB. In addition, the findings of this study have important implications for policy makers to design and implement the appropriate public health strategies and programs.

## Conclusions

This study revealed that the overall knowledge of Lesotho’s female respondents about TB was adequate while that of its male respondents was inadequate. Addressing the misconceptions and the identified associated factors with the knowledge of the general population of Lesotho about TB is an essential intervention. The strategies to improve the knowledge of Lesotho’s people about TB should focus on males, younger residents, those who are illiterate, are unmarried and are farmers. Special attention should be given to males, young residents, rural residents, residents who are illiterate and farmers to improve their attitude towards TB in Lesotho.

## Abbreviations

HIV: Human Immunodeficiency Virus
LDHS: Lesotho Demographic and Health Survey
MDR –TB: Multidrug Resistant Tuberculosis
MTB: Mycobacterium Tuberculosis
TB: Tuberculosis
USAID: United States Agency for International Development
WHO: World Health Organization

## References

[1] WHO, Global Tuberculosis Report. 2014. Available at: http://www.who.int/iris/handle/10665/137094. Accessed 15 Sept 2016.

[2] WHO, Global Tuberculosis Report 2015. Available at: http://www.who.int/iris/handle/10665/191102. Accessed 2 Dec 2016.

[3] WHO, Global tuberculosis report. 2016. Available at: http://apps.who.int/medicinedocs/documents/s23098en/s23098en.pdf. Accessed 2 June 2017.

[4] WHO, Estimates of TB and MDR-TB burden, generated: 2017–03. Available at: https://extranet.who.int/sree/Reports?op=Replet&name=%2FWHO_HQ_Reports%2FG2%2FPROD%2FEXT%2FTBCountryProfile&ISO2=LS&LAN=EN&outtype=html. Accessed 10 Sept 2017.

[5] Ministry of Health (MOH), Global AIDS Response Progress Report. 2015. Available: http://www.unaids.org/sites/default/files/country/documents/LSO_narrative_report_2015.pdf. Accessed 20 Sept 2017.

[6] Farhanah AW, Sarimah A, Jafri Malin A, Hasnan J, Siti Suraiya MN, Wan Mohd Zahiruddin WM (2016). Updates on knowledge, attitude and preventive practices on tuberculosis among healthcare workers. Malays J Med Sci.

[7] Bhebhe L, Vanrooyen C, Steinberg GW (2014). Attitudes, Knowledge and practices of healthcare workers regarding occupational exposure of pulmonary tuberculosis. African J Prim Health Care Fam Med.

[8] Malangu N, Adebanjo OD (2015). Knowledge and practices about multidrug-resistant tuberculosis amongst healthcare workers in Maseru. Afr J Prm Health Care Fam Med.

[9] Moselinyane L., Knowledge, attitudes and perceptions of TB non-adherent and adherent 2–3 years after their initial registration at Botšabelo clinic, Maseru, Lesotho. 20111. Available: http://etd.uwc.ac.za/xmlui/handle/11394/5029. Accessed 30 June 2017.

[10] Dr. Arinze Okolo, Knowledge, attitude and practice of non-dental health care providers in relation to the oral manifestations of HIV/AIDS in Butha-Buthe district, Lesotho. 2016. http://etd.uwc.ac.za/xmlui/handle/11394/5029. Accessed 10 July 2017.

[11] Ministry of Health [Lesotho] and ICF International.. Lesotho Demographic and Health Survey- 2014. 2016. Available: https://dhsprogram.com/publications/publication-fr309-dhs-final-reports.cfm. Accessed 24 Mar 2017.

[12] Population Reference Bureau, World population data sheet. 2016. Available: https://www.prb.org/wp-content/uploads/2016/08/prb-wpds2016-web-2016.pdf. Accessed 1 June 2017.

[13] A. O. Hassan, Richard Olukolade, Q. C. Ogbuji, et al., “Knowledge about tuberculosis: a precursor to effective TB control—findings from a follow-up national KAP study on tuberculosis among Nigerians,” Tuberc Res Treat, 2017, Article ID 6309092, 8, 2017. doi:10.1155/2017/6309092. 10.1155/2017/6309092 PMC5624149 29075531

[14] Anochie PI, Onyeneke EC, Onyeozirila AC, Igbolekwu LC, Onyeneke BC, Ogu AC (2013). Evaluation of public awareness and attitude to pulmonary tuberculosis in a Nigerian rural community. GERMS.

[15] Uchenna OU, Chukwu JN, Oshi DC, Nwafor CC, Meka AO (2014). Assessment of tuberculosis-related knowledge, attitudes and practices in Enugu, South East Nigeria. J Infect Dis Immun.

[16] Das P, Basu M, Dutta S, Das D (2012). Perception of tuberculosis among general patients of tertiary care hospitals of Bengal. Lung India.

[17] Gilani SI, Khurram M (2012). Perception of tuberculosis in Pakistan: findings of a nation-wide survey. J Pak Med Assoc..

[18] Silva LS, de Sousa JGD, de Paiva LO, Roberto O, Pinheiro LF (2016). Knowledge about tuberculosis among Brazilians. J Clin Respir Dis Care.

[19] Vidhani M, Vadgam P (2012). Awareness regarding Pulmonary Tuberculosis - A Study among Patient Taking Treatment of Tuberculosis in Rural Surat, Gujarat. Scope Med.

[20] Kilale AM (2008). Perceptions of tuberculosis and treatment seeking behaviour in Ilala and Kinondoni municipalities in Tanzania. Tanzan J Health Res.

[21] Legesse (2010). Knowledge and perception of pulmonary tuberculosis in pastoral communities in the middle and lower Awash Valley of Afar region, Ethiopia. BMC Public Health.

[22] Tolossa (2014). Community knowledge, attitude, and practices towards tuberculosis in Shinile town, Somali regional state, eastern Ethiopia: a cross-sectional study. BMC Public Health.

[23] Bati J, Legesse M, Medhin G (2013). Community’s knowledge, attitudes and practices about tuberculosis in Itang Special District, Gambella Region, South Western Ethiopia. BMC Public Health.

[24] Nyasulu P (2016). Knowledge and perception about tuberculosis among children attending primary school in Ntcheu District, Malawi. J Multidiscip Healthc.

[25] Hibstu DT, Bago BJ (2016). Knowledge, attitude and practice of tuberculosis and its transmission among high school students in Yirgacheffe town, Gedeo zone, southern Ethiopia. J Infect Dis Preve Med.

[26] Renuka, Ms.D.Karthika and Murali Dhar, Knowledge and Awareness of Tuberculosis among High School Students of Mysore City. Al Ame en J Med S c i (2012)5 (3 ) :333–336.

[27] Agho KE (2014). Determinants of the knowledge of and attitude towards tuberculosis in Nigeria. J Health Popul Nutr.

[28] Sifrash Meseret Gelaw, “Socioeconomic factors associated with knowledge on tuberculosis among adults in Ethiopia,” Tuberc Res Treat 2016, Article ID 6207457, 11 2016. doi:10.1155/2016/6207457. 10.1155/2016/6207457 PMC4753341 26949546

[29] Obuku EA (2012). Socio-demographic determinants and prevalence of tuberculosis knowledge in three slum populations of Uganda. BMC Public Health.

[30] Jurcev-Savicević A (2011). towards tuberculosis and sources of tuberculosis-related information: study on patients in outpatient settings in Split. Croatia..

[31] Mweemba P, Haruzivishe C, Siziya S, Chipimo PJ, Cristenson K, Johansson E (2008). Knowledge, Attitude and Compliance with Tuberculosis Treatment, Lusaka, Zambia. Med J Zamb.

[32] Tasnim S, Rahman A, Hoque FMA (2012). Patient’s knowledge and attitude towards tuberculosis in an urban setting. Pulm Med.

